# Biomass from microalgae: the potential of domestication towards sustainable biofactories

**DOI:** 10.1186/s12934-018-1019-3

**Published:** 2018-11-10

**Authors:** Manuel Benedetti, Valeria Vecchi, Simone Barera, Luca Dall’Osto

**Affiliations:** Dipartimento di Biotecnologie, Università di Verona, Strada Le Grazie 15, 37134 Verona, Italy

**Keywords:** Microalgae, Photosynthesis, Photobioreactor, Biomass, Bio-based products, Light-use efficiency, Strain domestication, Molecular genetic

## Abstract

Interest in bulk biomass from microalgae, for the extraction of high-value nutraceuticals, bio-products, animal feed and as a source of renewable fuels, is high. Advantages of microalgal vs. plant biomass production include higher yield, use of non-arable land, recovery of nutrients from wastewater, efficient carbon capture and faster development of new domesticated strains. Moreover, adaptation to a wide range of environmental conditions evolved a great genetic diversity within this polyphyletic group, making microalgae a rich source of interesting and useful metabolites. Microalgae have the potential to satisfy many global demands; however, realization of this potential requires a decrease of the current production costs. Average productivity of the most common industrial strains is far lower than maximal theoretical estimations, suggesting that identification of factors limiting biomass yield and removing bottlenecks are pivotal in domestication strategies aimed to make algal-derived bio-products profitable on the industrial scale. In particular, the light-to-biomass conversion efficiency represents a major constraint to finally fill the gap between theoretical and industrial productivity. In this respect, recent results suggest that significant yield enhancement is feasible. Full realization of this potential requires further advances in cultivation techniques, together with genetic manipulation of both algal physiology and metabolic networks, to maximize the efficiency with which solar energy is converted into biomass and bio-products. In this review, we draft the molecular events of photosynthesis which regulate the conversion of light into biomass, and discuss how these can be targeted to enhance productivity through mutagenesis, strain selection or genetic engineering. We outline major successes reached, and promising strategies to achieving significant contributions to future microalgae-based biotechnology.

## Background

### Microalgae, a promising feedstock option

Approximately 100,000 terawatts-year (TW-y) power from sunlight reach the surface of our planet. This is a renewable resource exceeding the current human global energy demand (15 TW-y) and the 24 TW-year predicted for anthropic activities by 2030 [1, 2] by > 3 orders of magnitude. Sunlight might fully provide for future world energy demand [3] and yet its dilute nature represents a major challenge for concentrating, harvesting, storing it efficiently. Oxygenic photosynthesis converts CO_2_ into reduced carbon compounds using light and water; through this process photoautotrophic organisms, namely plants, algae and cyanobacteria, store solar energy at a rate of 120 TW-y at the global scale. Therefore, using sunlight and CO_2_ to produce a variety of organic molecules and biomass, by the extensive cultivation of photosynthetic organisms, has the potential to cover a significant portion of global energy demand [4], besides providing for an effective CO_2_ capture from e.g. power plants or other large-scale emission sources. As unique feature, photosynthesis allows for direct energy storage into liquid fuels which can be used in the existing transport systems, while other forms of renewable energy such as hydro-, wind or photovoltaic power, cannot.

Thus, mass culture of microalgae has gained interest in the past few decades. Indeed, beside small-scale traditional cultivations mainly aimed to human feeding, commercial production of algae on a larger scale has been identified in recent years as a renewable and environmentally sustainable strategy for feedstock production. Microalgae include a wide group of photosynthetic, eukaryotic, unicellular organisms: green microalgae, belonging to the class Chlorophyceae, include genera which are among the most widely used for industrial applications, such as *Haematococcus*, *Chlorella* and *Dunaliella*. Diatoms and cyanobacteria, which also represent a valuable biotechnological platform [5, 6], will not be included in this review.

The phyletic group of green microalgae include species which have adapted to diverse environmental conditions, even extreme, of the planet [7]. By considering that the unclassified species likely represent the majority of this group [8], it comes that green microalgae are a source of metabolic and genetic diversity [9]. Microalgal biomass represents an energy-rich feedstock, which received increasing attention for commercial cultivation in open ponds or closed photobioreactors (PBRs). So far industrial applications include production of bioactive compounds [10], recombinant proteins [11], next generation biofuels and wastewater treatment [12]. Once target products extracted, the residual biomass can be further processed into livestock feed, organic fertilizer and biostimulants, or used for energy cogeneration [13–15]; therefore, biorefinery processes applied to mono-species cultivation can yield a large variety of resources.

Although photosynthetic machinery is similar to that of plants, microalgae convert solar energy into biomass and fix CO_2_ at efficiencies that are appreciably higher than land plants [16]. The maximal conversion efficiencies of solar radiation into biomass are 4.6% for C3 plants and 6.0% for C4 plants at 30 °C, which drops to 2.9% and 4.2% respectively, when measured in the field [17].

Theoretical quantification of 8–10% in energy conversion efficiency of microalgae [18] translates into an expected maximal productivity of 280 ton of algal biomass hectare (ha)^−1^ year^−1^, while outdoor mass cultivation record yield beyond 100 ton ha^−1^ year^−1^ could not be reached [19]. This compares to 0.2% of energy conversion efficiency and an average of 10 ton ha^−1^ year^−1^, reported for sugarcane field trials in the tropics. When considering oil yield extracted from plant vs. algal biomass, palm oil can produce a maximum of 4–5 ton ha^−1^ year^−1^ [20, 21] vs. 30 ton ha^−1^ year^−1^ [19]. Thus, record yields of microalgal culture at temperate latitudes is > 5 times higher respect to the case of the best figure for a plant crop.

Multiple reasons contribute to such a feature:

i. When growing in aerated liquid cultures, cells have easy access to light, CO_2_ and nutrients. They lack non-productive (heterotrophic) organs to maintain, and the simpler unicellular structure make the whole biomass fully photosynthetically active, irrespective to seasonal life cycle;
ii. Algae are metabolically flexible and have a short doubling time. Although most microalgae are primarily photoautotrophs, many species undergo metabolic shift to heterotrophy upon changes in environmental conditions, utilizing organic compounds as C and energy source or to mixotrophy (carrying out photosynthesis as the main energy source, and both organic molecules and carbon dioxide are used as C source). Moreover, under optimal growth conditions, most species have doubling time of a few hours, and cultures reach as much as 10 g l^−1^ of heterotrophic dry weight (DW) biomass and 6 g l^−1^ of photoautotrophic DW biomass [22, 23];
iii. Microalgae do not require fertile land, and can grow in wastelands, using brackish or waste water, or even sea water in the case of marine species; hence, their cultivation does not compete with resources for conventional food production, and would be more environmentally sustainable respect to extensive cultivation of crops. Therefore, microalgae offer the opportunity to shift part of unsustainable farming and fishing routines toward unproductive region;
iv. Different species can be selected for specific growth conditions, suited to the local climate, which is more difficult with conventional crops.

## Main text

### The most promising microalgae species for production of valuable compounds and for biotechnology applications

In this paragraph, the bio-technological applications of best wild type species are reviewed with focus on high-value production chemicals and biomass for biofuels (Table 1). Currently, the most relevant microalgal species for high-value chemicals production are the cyanobacterium *Arthrospira platensis* (formerly known as Spirulina) and the green microalgae *Chlorella vulgaris*, *Dunaliella salina* and *Haematococcus pluvialis*, which are mainly dedicated to the production of single products in large-scale cultivation systems.

**Table 1:** Noteworthy microalgae species and their biotechnological applications for production of high-value metabolites

Application	Industrial and medical field	Cosmetics and food colorant	High-value metabolites	Biofuel	Dietary supplement and nutraceuticals
*Bioproduct category* *Microalgae species* (metabolite)	*Polysaccharides* *Chlorella* spp. (*β*-*glucans, starch*) *Porphyridium cruentum* *Netrium digitus* *Phycotoxins* *Amphidinium* *Dinophysis* *Prorocentrum* *Phycobiliproteins* Red algae (Phycoerythrin)	*Phycobiliproteins* *Arthrospyra platensis* (phycocyanin) *Carotenoids* *Arthrospyra platensis* *Chlorella vugaris* *Haematococcus pluvialis* *Chlorella zofingiensis* (astaxanthin) *Dunaliella salina* (β-carotene)	*Mycosporine-like amino acids* *Aphanizomenon flos*-*aquae* *Vitamins* *Euglena gracilis* (biotin, α-tocopherol) *Prototheca moriformis* **(**ascorbic acid) *Arthrospyra platensis* *Chlorella* spp. *Proteins* *Arthrospyra platensis* *Chlorella* spp.	*Oil to biodiesel* *Botryococcus braunii* *Chlorella* spp. *Dunaliella salina* *Monoraphidium* *contortum* *Scenedesmus* spp. *Carbohydrate to bioethanol* *Spirogyra* spp. *Chlorococum* spp. *Bio-hydrogen* *Chlamydomonas reinhardtii*	*Polyunsaturated fatty acids (PUFAs)* *Parietochloris incise* *Porphyridium* spp. (arachidonic acid) *Arthrospyra platensis* *Rhodomonas salina* *Tetraselmis uecica* (α-linolenic acid) *Chlorella minutissima* *Monodosus* spp. *Nannochloropsis* spp. *Neochloris oleoabundans* *Pavlova lutheri* (eicosapentaenoic acid) *Crypthecodiuimu* spp. *Isochrysis galbana* *Schizochytrium* spp. *Thalassiosira* spp. *Thraustochytrium* spp. (docosahexaenoic acid)

*Arthrospira platensis* is exploited as source of nutraceuticals [24], long-chain polyunsaturated fatty acids (lc-PUFAs) [25], carotenoids [26] and proteins [27]. Other applications of Spirulina are in the medical field as a therapeutic [28, 29], as antioxidant [25] and for the extraction of the blue pigment phycocyanin, approved as food colorant by FDA [30].

Genus *Chlorella* includes a number of species which are widely commercialized for production of nutraceuticals. Besides to the high protein, carotenoids and vitamins content [31], *C. vulgaris* contains also β- and α-glucans, d-glucose polysaccharides which act as immune stimulators, free-radical scavengers and anti-cancer compounds [32]. Moreover, *Chlorella* has been successfully used to produce starch, reaching 26% DW yield under mixotrophic condition [33].

Carotenoids represent the commercial product from microalgae with highest success. Carotenoids are widely used as food colorants, aquaculture feed additives and components for cosmetics and skin care; carotenoids also have biomedical applications, including anti-inflammatory activities which are related to their strong antioxidant properties [34]. β-carotene, the first carotenoid successfully marketed at large scale, is produced from the halophilic alga *Dunaliella salina* through both extensive cultivation in ponds and intensive cultivation in PBRs [35]. Recently, new strains with different ability to accumulate carotenoids and different capacity of photoprotection against high light stress have been isolated; the most promising strain was characterized by a β-carotene productivity of 3.5 g l^−1^ day^−1^ at 1500 µmol m^−2^ s^−1^ [36]. Currently, various strains of *D. salina* growing at different salinity conditions are available [37].

Astaxanthin is a high-value, red keto-carotenoid, successfully commercialized by many companies worldwide through cultivation of the green alga *Haematococcus pluvialis*. Under various stress conditions, this alga changes from a thin-wall mobile phase to a red thick-wall resting phase, in which astaxanthin can reach up to 5% DW [38]. Astaxanthin is widely employed in the feed, cosmetic, aquaculture, nutraceutical and pharmaceutical industries because of its antioxidant potential [39]. Moreover, astaxanthin-rich *Haematococcus* is a popular nutraceutical antioxidant for human diet [40]. *Chlorella zofingiensis* has been proposed as an alternative astaxanthin source which is more reliable in growth [41].

Biosynthesis of fatty acids and triglycerides (TAGs) is relevant for several industrial applications. Microalgae are the primary producers of lc-PUFAs such as eicosapentaenoic acid (EPA) and docosahexaenoic acid (DHA), which accumulate in the oil of many fish species. Aquaculture farming increased demand of lc-PUFAs for nutrition, which are currently produced from fish oil, while a more sustainable lc-PUFA supply is seeked. Several marine algal species are rich in lc-PUFAs thus have a great potential for biorefinery: these include *Thraustochytrium* sp., *Pavlova lutheri*, *Nannochloropsis gaditana*, *Isochrysis galbana*, *Crypthecodinium cohnii* (rich in DHA and EPA), *Rhodomonas salina* and *Tetraselmis suecica* (α-linolenic acid) [42, 43] and *Parietochloris incisa* (arachidonic acid) [44]; these are currently exploited by a number of small companies, marketing biomass of high-values but at small-scale. Lc-PUFAs are important elements for human diet: DHA plays a crucial role as anti-inflammation molecule in allergic diseases and has considerable benefits on visual and cognitive functions; optimization of the ratio of lc-PUFAs in nutraceuticals may contribute to reduce the severity of allergic disease symptoms [45]; oil from *Nannochloropsis*, *Rhodomonas* and *Tetraselmis* has higher antioxidant properties respect to fish oil, likely due to the content of valuable carotenoids and polyphenols and is expected to replace fish oils in diets soon [46].

Further species with high potential for large-scale exploitation of extracellular polysaccharides include the microalgae *Porphyridium* and *Desmidiales* spp. Red microalgae *Porphyridium* spp. are fast-growing and accumulate extracellular polysaccharides commercially used in cosmetic and medical field [47, 48]. Amongst *Desmidiales*, *Netrium digitus* has been successfully cultivated in porous substrate bioreactor, reaching a maximum product concentration of 25 g m^−2^ [49].

In the last decade, microalgae have received increasing interest as a source of biomass for replacing fossil fuels. Liquid fuels derived from raw biomass are an attractive source of renewable energy, to be used in transport system or energy cogeneration. With respect to the major biofuels currently produced worldwide, namely bio-ethanol from sugar cane and biodiesel from oil crops, the so called “third generation” (microalgal) biofuels, are considered as a promising option, since these organisms are highly productive and provide a solution for the food *vs*. fuel problem [50].

Green algae accumulate high levels of polysaccharides both as cell-wall constituents and storage molecules that can be fermented to bioethanol [51, 52]. Oil fraction of algal biomass, which range from 20 to 60% DW depending on the species and growth conditions [19, 53], is processed by transesterification to produce biodiesel. In this respect, promising species belong to the genus *Chlorella* [54], *Scenedesmus* [55] and *Monoraphidium*, the latter showing high productivity and high-quality lipid profile [56]. An unusually rich source of TAGs is the green microalga *Botryococcus braunii* (hydrocarbons constituting up to 75% of its DW), however its potential is limited by the slow growth [57].

Despite great advantages offered by microalgae exploitation, production of third generation biodiesel is still far from being commercially viable [58]. As an alternative, biogas generation through anaerobic digestion of microalgal biomass has been proposed as a more energetically-favorable process [59]. The efficiency of biogas production is species-dependent because is based on cell wall degradability and sometime limited by the content in molecules inhibiting growth of methanogenic *Archaea* [60]. A number of pre-treatment procedures have been tested, including cell wall disruption by chemical/physical methods or enzymatic hydrolysis, which enhanced bio-methane yield [14, 61, 62].

Finally, microalgae and cyanobacteria can produce bio-hydrogen through photo-fermentation, in an anaerobic process involving oxidation of ferredoxin by the hydrogenase enzyme [63, 64]. Although biological H_2_ shows great promise for generating future, large scale sustainable energy, a number of bottlenecks still limit its production [65]; however, recent result [66] identified in *C. reinhardtii* promising targets for genetic engineering of H_2_ production capacity while the use of temperature-sensitive conditional PSII mutants has been proposed in order to separate the oxygenic biomass-accumulating phase from the oxygen sensitive hydrogenase activity [67].

Microalgae are gaining importance in the biological offset of polluted matrix, because of their ability to thrive under extreme or polluted condition: they serve for direct carbon capture, a way for reducing CO_2_ released by large-scale emission plants [68]. Promising species include *Scenedesmus obliquus*, *Chlorella vulgaris*, *Chlorella protothecoides* and *Spirulina* spp., which can grow up to 15–18% CO_2_, although highest productivity was observed around 10% CO_2_ [69, 70]. In last years, *N. gaditana* is arising as promising species for CO_2_ removal due to a high biofixation rate—more than 1.7 g^−1^ l^−1^ day^−1^ [71].

Growth in open ponds is an established technology for bioremediation of wastewater and nutrient recovery in the form of biomass [12]. The effectiveness of microalgae to use inorganic N and P to sustain growth as well as their capacity to sequester heavy metals and toxic compounds, have been demonstrated with a wide range of wastewaters, and at a range of scales [72, 73].

### Technical challenges to cost-effective, large-scale microalgae production

Despite a number of industrial applications of microalgae have been proposed and studied in lab-scale, the only successful commercial exploitation of microalgal mass culture is the production of carotenoids, namely β-carotene by *D. salina*, and astaxanthin by *H. pluvialis* [10]. Other species (*Chlorella* spp., *Spirulina* spp.) produce high-value compounds, however these productions are currently applied at small-scale cultivations, which need significant reduction in operating costs to become competitive with the same molecules extracted from other feedstocks [74]. A number of species have been identified as promising targets for biorefinery approaches [75], which, however, have not yet come to economic viability. Thus, while microalgae represent a promising source of valuable bio-based products, (1) optimization of both cultivation and processing technologies, together with (2) selection of candidates with high growth rate and cell density, are required to make the process profitable [76]. An overview of the approaches and the major challenges related to point (1) are presented as follows.

In algal biomass pipeline, there are many elements which contribute to the overall cost of the process. Major factors to take into account are (i) the choice of production system, (ii) the strategies to supply nutrients, aeration, mixing, and (iii) how to harvest and process biomass, and (iv) the procedure to avoid infections and contaminants. Different approaches are available, each having benefits and limitations [77].

Microalgae are mainly cultivated in open ponds, which are cheaper to build, and easier to operate and to scale-up than closed systems. Generally, ponds are raceways at depth of 20–30 cm, in which biomass is mixed by paddles or left unstirred. Drawbacks of these systems include the complication of controlling contaminations, and the difficult of keeping constant growth parameters (e.g. temperature, pH, light); moreover, they suffer of low productivities (< 20 g m^−2^ day^−1^) due to poor gas exchange and dark zone, therefore low cell density forces to cover extensive areas and requires high costs for harvesting the biomass.

As an alternative to open ponds, closed PBRs allow for higher productivities (~ 0.8–1.5 g l^−1^ day^−1^ [19], up to 10 times higher than ponds). Lab-scale PBRs include flat reactors, tubular reactors or vertical plastic bags, with more control over the growth environment, in which biomass is mixed by air-lift or by pumping. These configurations allow higher cell density than ponds, thus improve economic viability of production, however they (i) have high building costs and are difficult to scale-up, (ii) can operate in a sterile mode, which however adds to the management fees, and (iii) require a high energy input for gas exchange [78, 79]. Together with light, CO_2_ and nutrients must be supplied to maximize the growth rate, and it significantly affects the economy balance. CO_2_ can be delivered through direct bubbling, and its distribution in the culture represents an additional cost factor; another challenge is the removal of excess O_2_ which, above air level, inhibits photosynthesis [80].

A third production method is the surface-attached algal biofilm, which showed greater yield than suspended culture, and lower land and water requirements, in lab-scale trials. Algal biofilm system thus appears a good option for low-cost productions [81, 82], however more research is needed to move from bench-scale to pilot plant.

Following growth, biomass must be (i) harvested and (ii) processed to dryness. Both steps remain a major obstacle to industrial scale processing and contribute to ~ 1/3 of the final biomass cost. Current harvesting methods include chemical, mechanical and bio-based procedures: electrolytes or polymers are added to flocculate cells; centrifugation or flow filtration are rapid methods, which however implies high investments and operating costs; biological-based methods include auto-flocculation (at high pH, in excess of Ca^2+^ ions), bio-flocculation (caused by secreted polymers) or microbial-induced flocculation [83]. Dewatering and drying of biomass is required, and the low biomass concentration (0.1–1% w/w) affects the cost of the final product [84]. In case the release of the products from cells is required, it should be as more energy-efficient as possible, avoiding the use of expensive solvents, and costs for treating biomass should be minimized. Novel approaches which limit the use of solvents, e.g. based on enzymatic hydrolysis of the cell wall [51, 61], still suffer for expensive enzyme production. To significantly reduce energy penalty of the production process, the waste biomass can be directed to anaerobic digestion and production of biogas, fertilizers, soil amendments or feeds [13, 62, 85].

**Fig. 1:**
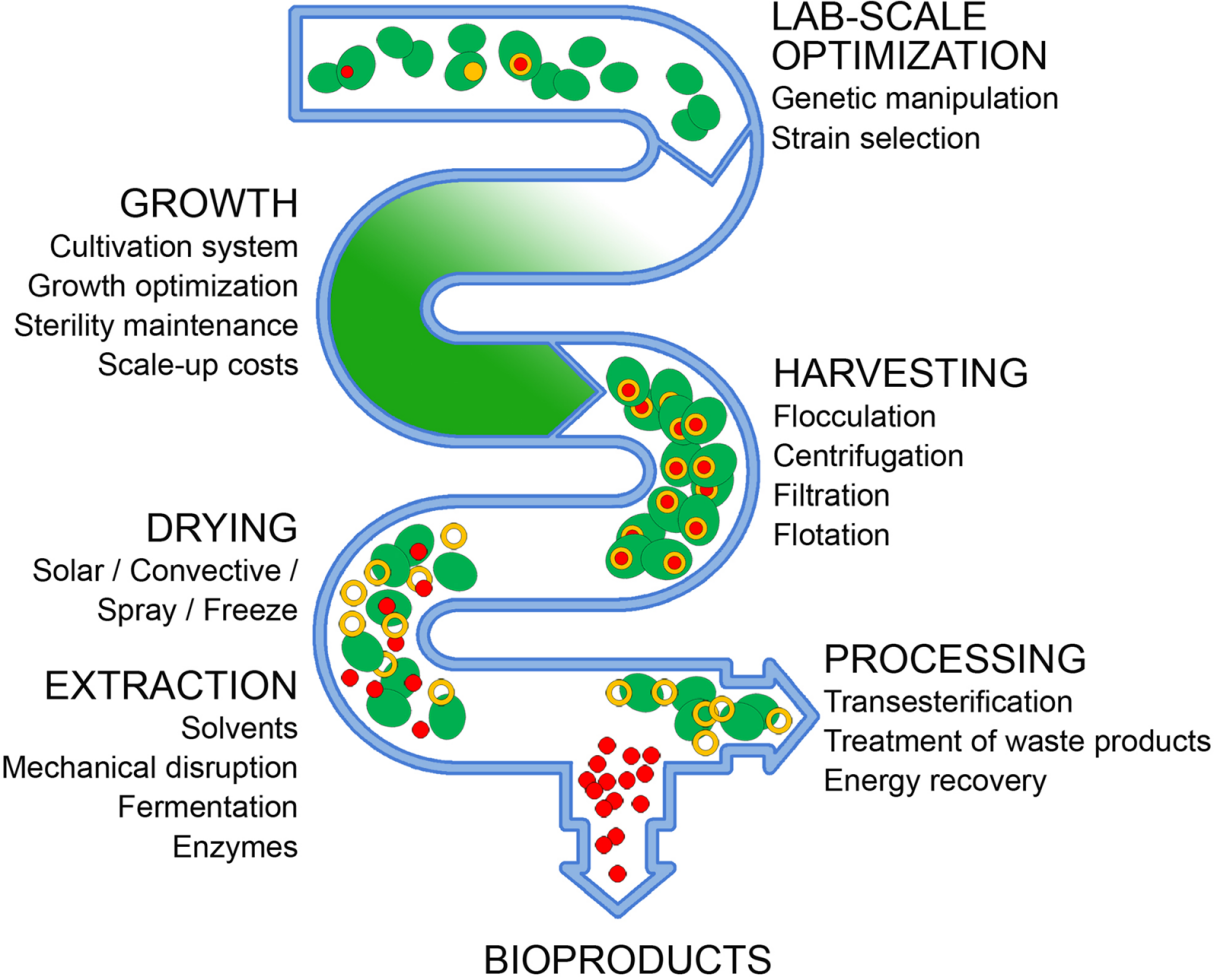
General scheme of the algal production chain. A number of factors, including the high cost of the infrastructure and the energy required for growth, harvesting and processing the algal biomass, significantly contribute to the cost of the whole production pipeline

Figure 1 shows the different stages in the production of algal biomass, including the factors to be considered and optimized, which contribute to the price of bio-products. In consideration of the processing costs at the present state of technology, engineering optimization is necessary to find new, cost-effective methods of producing large quantities of feedstock. However, integration of innovative technical solutions with strains improved by biotechnological approaches, appears essential.

The overall cost of a biomolecule is the results of cellular content of the desired product, and growth rate of the culture, the latter being dependent on the efficiency at which photosynthetically active radiation (PAR) is used to drive photosynthesis. Indeed, an area of promising research aims at improving the light-to-biomass conversion efficiency under mass culture conditions.

### Biological constraints in light-to-biomass conversion efficiency

Calculations in [18] provided both theoretical maxima of solar energy conversion efficiencies in photosynthesis and productivity yield of microalgae, equal to 8–10% solar-to-biomass and 280 ton of dry biomass ha^−1^ year^−1^, respectively. Instead, outdoor mass cultivation showed that, with the present technology and wild type strains, annual productivities beyond 80–100 ton DW ha^−1^ year^−1^ cannot be maintained at large scale and over long periods [19]. Overcoming this gap, which limits exploitation of microalgae to their full potential, is therefore essential.

Wild type algal strains suffer of light use inefficiency. Enhancing light-to-biomass conversion efficiency will help counterbalancing the cost of energy and nutrients used in the cultivation system, as well as reducing the costs of downstream biomass processing, making it a target for genetic improvement.

**Fig. 2:**
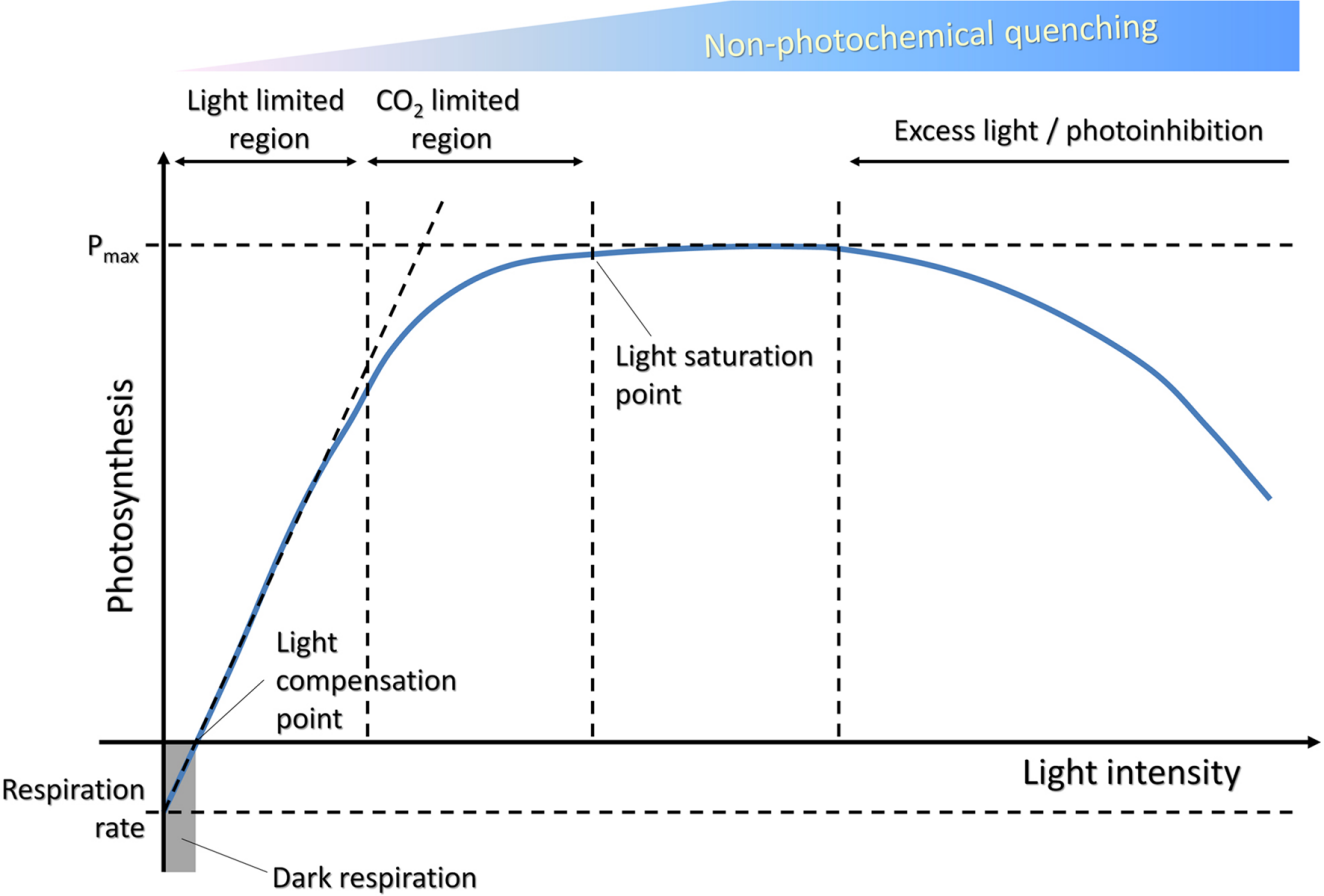
Light response curves for photosynthesis. The light compensation point is the minimum light intensity at which the organism shows a gain of carbon fixation. The net photosynthetic rate shows a linear rise in response to increased light, in the range of light limitation. At higher light levels, saturation occurs as the efficiency of the photosynthetic mechanism is reduced due to the activation of energy quenching processes. Under excess light conditions, net photosynthesis can decline as a result of photoxidative stress

Regulation of light harvesting capacity is crucial for cells in order to balance light reactions and downstream biochemical events of photosynthesis. Indeed, autotrophs have evolved regulatory mechanisms, to fine-tune continuous transitions between “conservative” and “dissipative” state of absorbed energy. In particular, photosynthesis typically displays a light saturation curve (Fig. 2), in which 3 distinct phases can be identified: (1) at low irradiance, namely when light is the limiting factor, the photosynthetic rate increases linearly with light intensity; (2) at increasing irradiances, the limiting factor becomes CO_2_ fixation rate, thus photosynthetic rate increases non-linearly as a function of light; (3) when light intensity overcomes the rate of downstream biochemical reactions, photosystems get rid of energy absorbed in excess, and in this phase photosynthetic rate reaches a plateau.

In a dilute culture of *C. vulgaris*, where light attenuation is minimized, light saturation is reached at around 1200 µmol photons m^−2^ s^−1^. At this irradiance, algae protect themselves from excess illumination by triggering the non-photochemical quenching (NPQ) mechanism, a feedback-regulated de-excitation of Chls that operates in the PSII, to prevent over-excitation of reaction centers (Fig. 2). Although light-dependent energy quenching is a property of all photosynthetic organisms, large differences in amplitude and kinetics can be observed. Some microalgae, such as *C. zofingiensis*, exhibit constitutively high energy quenching activity [86], while in other species (e.g. *C. reinhardtii*) energy quenching is significantly activated only upon acclimation to excess light conditions [87]. NPQ activates when light excitation flux exceeds CO_2_ fixation rate. However, algae can experience very high light intensity, saturating photoprotective mechanisms. Light in excess of photosynthesis saturation level is dissipated rather than contributing to biomass accumulation, or even causes synthesis of reactive oxygen species (ROS), which damage the photosynthetic machinery and affect biomass yield. *C. reinhardtii* mutant *npq4*, devoid of NPQ response, was indeed more susceptible to photo-oxidation [87].

Due to the absorption of pigments bound to the large antenna systems in both photosystems, light distribution within the culture is inhomogeneous, and this strongly contributes to the gap between theoretical vs. real productivity. Such large arrays of antenna complexes have been selected by evolution as favorable trait, since they maximize the light-harvesting capacity and therefore the adaptation to a natural water environment where light is often scarce and limits growth, and cell density remains low. Contrary to the natural environment, growth conditions in mass cultures requires high cell biomass per volume of installed facility. However, this results in high optical density and light shortage in the deeper layers of the culture while cells at the surface layers intercept most photons, resulting in saturation of photosynthesis, dissipation of excess energy and/or photoinhibition. The most inner layers easily drop below the compensation point of photosynthesis while active respiration consumes energy. Thus, dense algal cultures suffer both photo-deprivation and photo-inhibition, decreasing the overall light-to-biomass conversion efficiency far below the theoretical score. Rapid mixing of biomass is often suggested as a solution to light gradients, but it is not: rapid light/dark cycles between dark and over-saturating irradiances have a deleterious effect on biomass yield [88]. Modeling of the light-response curve of photosynthesis in a culture system [89] suggests that optimal setting of OD in the culture limits shading while maximizes light absorption and net photosynthesis.

One additional factor which contributes to the inefficiency of photosynthesis is carbon fixation: the enzyme RuBisCO, which catalyzes the carboxylation of Ribulose 1,5-bisphosphate (RuBP), has low affinity for CO_2_ and can also use O_2_ to give oxygenated substrates [90], which ultimately results in ATP/NADPH consumption and loss of fixed C. To compensate for this, RuBisCO accumulates to as much as 50% of total soluble proteins of the cell. Inefficient light use in mass culture, due to inhomogeneous light distribution, results from limitations in the turnover rate of the Calvin-Benson cycle. The selection of strains with higher RuBP carboxylation activity would therefore be a major goal for the optimization of photosynthesis. Possible strategies include the heterologous expression of variants of RuBisCO with a higher specific activity, or the control of allosteric regulators (e.g. RuBisCO activase) to yield into suppression of oxygenase activity.

Interestingly, many microalgae use biophysical carbon concentrating mechanisms for active retention of inorganic C [91] to increase CO_2_ availability at the RuBisCO active site within the pyrenoid, a micro-compartment of the chloroplast. Engineering such a mechanism into algal species devoid of pyrenoids might augment the overall C fixation rate and thus photosynthetic efficiency.

In conclusion, results reported in this section suggest that modulation of photosynthetic reactions is a key factor controlling biomass yield at both saturating and sub-saturating irradiances, that is worth to be considered for a domestication strategy aimed at improving performances in PBRs.

### Promises of domestication by forward genetic in improving photosynthetic efficiency

According to the previous section, a gap between theoretical and real biomass productivities of microalgae originates from the high OD of cells. With respect to the problem of inhomogeneous light distribution, high density planting of crops is a condition limiting PAR penetration thus productivity, and it is not different than the condition of elevated cell density reachable in a PBR. Moreover, biomass production with wild type algal strains is poorly viable likely as farming with ancestral crop varieties.

The so called ‘Green Revolution’ of agriculture, a domestication based on breeding and phenotypic selection, succeeded in pursuing crop productivities over the past 50 years [92, 93]. Industrial application of microalgae may take advantage of a domestication approach, analogous to that carried out for modern crops. Thus, selection of strains carrying desired traits, together with implementing new alleles by random mutagenesis or genetic engineering, might improve performances in PBRs.

Random mutagenesis is recognized as a powerful technology in mutation breeding, widely employed for strain improvement and for studying the molecular basis of metabolic processes. Forward genetic approach is of particular relevance for algal biotechnology, since it avoids restrictions to GMO for outdoor production system [94]. The most common method for generating genetic variability in a population of microalgae is the mutagenesis induced by either physical methods (UV-light, γ- and X-rays) or chemical mutagens, e.g. *N*′-nitro-*N* nitrosoguanidine (NTG) and ethyl methanesulfonate (EMS).

Attempts for algae genetic improvement, aimed to enhance light-to-biomass conversion efficiency, relied on random mutagenesis and screening of favorable traits. These approaches, while overcame scarcity of genetic engineering tools in microalgae, needed for efficient screening strategies for strains with higher productivity. Some of these approaches, which succeeded in increasing photosynthetic yield, are presented as follows.

Due to detrimental effect of high OD for mass cultivation, strains carrying truncated antenna size were proposed to perform better in light transmittance than wild type [95]. Mutagenesis and screening of *C. reinhardtii* was employed to isolate mutants having a truncated light-harvesting system [96–100]: all of them showed a higher productivity than the wild type in bench-scale growth systems. Cazzaniga and collaborators [101] applied random mutagenesis to a thermotolerant, fast-growing strain of *C. sorokiniana*, and selected pale-green mutants by imaging Chl fluorescence. Mutants were able to perform photosynthesis more efficiently than wild type, minimizing photoinhibition in high light; the positive effect on photosynthetic productivity was confirmed in both lab-scale and outdoor PBRs. Similar results were obtained with *N. gaditana* strains having reduced cellular pigment content [102]. Finally, simultaneous knock-down of three light-harvesting complex proteins (LHCMB1, 2 and 3) in *C. reinhardtii*, by an RNAi triple knock-down strategy, resulted in improved light-to-H_2_ (+ 180% than wild type) and light-to-biomass (+ 165%) conversion efficiencies [103].

Implementation of biosynthetic pathway of accessory pigments, e.g. phycobilins or Chls, into genus of industrial interest, has been proposed for improving harvesting efficiency over the full PAR spectrum [4]. Recently, the enzyme responsible for the synthesis of Chl *f*, an oxidized form of Chl *a*, has been isolated from the cyanobacterium *C. fritschii* [104]; heterologous expression of Chl *f* synthase succeeded in accumulating this chromophore in *Synechococcus* sp. Since Chl *f* expands the spectral range for photosynthesis by absorbing far red light, its expression in microalgae may confer advantages for mass culture in PBRs, which suffers for detrimental sieve-effects at high cell densities. However, the feasibility of this approach, which assumes a correct binding of the new chromophores into the existing pigment-binding complexes, still await experimental confirmation.

CO_2_ fixation rate is a major limiting step in biomass yield, which arise from RuBisCO inefficiency. Genetic engineering of RuBisCO to increase its catalytic activity or to enhance its specificity towards CO_2_ have been proposed as ways to overcome these limitations [4]. It is worth noting that the natural diversity of RuBisCO is limited, likely because the interactions which support catalytic activity make most of the isoforms of this enzyme intolerant to mutations [90], indeed attempts to overcome its limitations by directed evolution, had scant success. Although variants of RuBisCO with higher activity have been identified [105, 106] their heterologous expression in algal strains of industrial potential is still missing. Recently, *E. coli*-based screen of new RuBisCO variants obtained by direct evolution allowed the identification of an unexplored subunit interface with potential of increasing CO_2_ fixation rate [107]. Site-directed mutagenesis in such subunit may generate novel variants whose enzymatic characteristics can be subsequently tested in microalgae in terms of enhanced CO_2_ fixation. An additional approach may reside in the generation of hybrid RuBisCO by using novel activase isoforms from chemolithoautotroph microorganisms such as *Acidithiobacillus ferrooxidans* [108].

A high photosynthetic efficiency is attainable only in low irradiance and controlled light environments, which allow most absorbed photons can be utilized by the culture; instead in the outdoor, efficiency drops due to fluctuating irradiances, exceeding the photosynthetic capacity. Autotrophs evolved mechanisms for regulating the efficiency of light capture, which can become target of domestication strategies. Several microalgal species e.g. *C. reinhardtii* and *H. pluvialis*, trigger fast phototactic response, for fine-tuning exposure to light. Indeed, phototaxis confers fitness advantage and it is regulated by cytoplasmic redox balance, which in turn is affected by photosynthetic electron transport rate [109]. In the attempt of isolating strains with improved light-use-efficiency, Kim and collaborators [110] analyzed a *C. reinhardtii* mutant population for rapid phototaxis response and identified mutants with enhanced photoautotrophic growth and lipid production, respectively 1.9- and 8.1-fold increases than wild type.

Photosynthetic organisms dynamically regulate the amplitude of NPQ (see “Biological constraints in light-to-biomass conversion efficiency” section): by balancing amplitudes of light harvesting vs. energy dissipation, they maintain optimal fitness in changing light environment. The slow relaxation rate of NPQ upon high- to low-light transition was considered to reduce the overall conversion efficiency of solar to biomass in microalgae, consistent with recent evidences in plants [111, 112]. Indeed, deletion of the OCP protein, responsible for NPQ response in cyanobacteria, resulted in a 30% higher biomass yield in mass cultures of *Synechocystis* than wild type cells [113]. Random insertional mutagenesis and Targeting Induced Local Lesions IN Genomes (TILLING) approach on *C. reinhardtii*, following by Chl fluorescence imaging screening, has produced mutants specifically devoid of *lhcsr* genes [87, 114]. In their report, [115] proposed that biomass productivity depends on LHCSR protein accumulation: *C. reinhardtii* strains lacking the two *lhcsr3* genes were more productive than wild type, thus confirming down-regulation of NPQ is a strategy for improving light use efficiency in microalgae. Instead, more recently, [116] observed no significant differences in biomass yield between *C. reinhardtii* wild type and the *npq4 lhcsr1* mutant, devoid of all Lhcsr isoforms.

Microalgae growing in mass culture experience rapid changes in the irradiance due to cell mixing into the PBR. The amount of time spent in sub-saturating vs. excess light influences the biomass productivity, which is lower in fluctuating light conditions [115] possibly due to the metabolic energy needed to repair photodamage. Hence, improving photosynthetic efficiency in excess light conditions is potentially a major goal for establishing efficient outdoor cultivation. Research efforts aimed to obtain non-GMO algal strain tolerant to excess light, mainly focused on the model alga *C. reinhardtii.* Förster et al. [117] isolated *very high light resistant* (VHL-R) mutations, which allowed near maximal growth rate at irradiances lethal to the control genotype; characterization of these strains reveals they affected the regulatory pathways which modulate photoprotective response, including PSII repair and ROS detoxification. In [118], wild type strain was UV-mutagenized and plated onto medium containing a lethal concentration of the photosensitizer Red Bengal; by this approach, SOR1 was identified as a factor enhancing tolerance to photooxidative stress conditions. Schierenbeck and coworkers [119] performed UV-mutagenesis followed by selection under high irradiance (2000 μmol m^−2^ s^−1^); the two mutations selected, which both mapped in the putative Light Responsive Signal 1 (LSR1) gene, conferred an improved resistance of cells against exogenous ROS. Recent results concern the isolation of pale-green, singlet oxygen resistant mutant by EMS-mutagenized *C. vulgaris,* which showed biomass yield enhancement by 68% than wild type strain (Dall’Osto et al. unpublished results).

### Improving algal biomass productivity by genetic engineering: methods, state of the art and perspectives

**Fig. 3:**
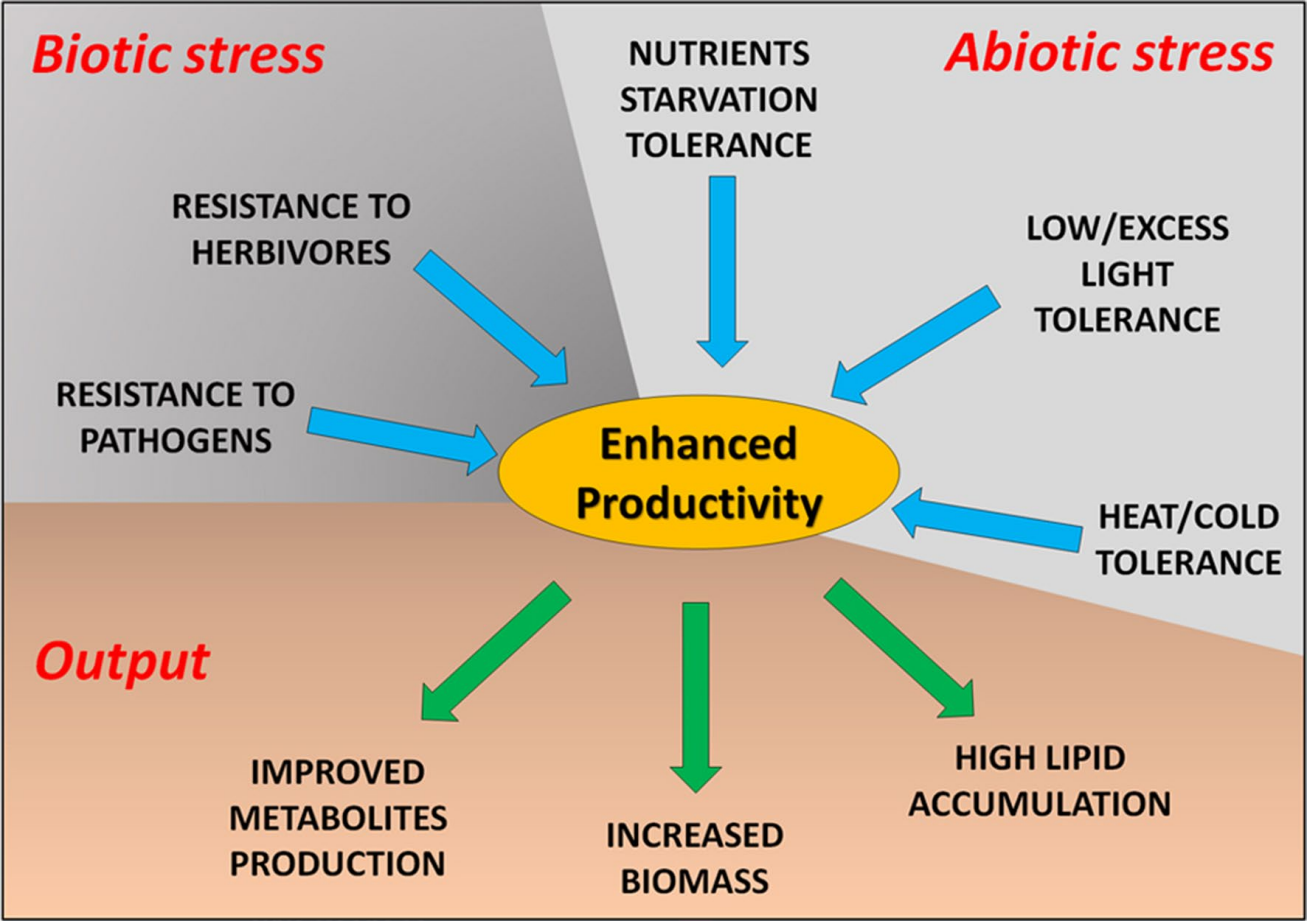
Schematic depiction of the major desirable traits to be either implemented or improved, toward higher productivity of microalgae in mass culture

Genetic manipulation approaches have the potential to revolutionize industry based on microalgae cultivation. These include transfer of genes isolated from other species to generate strains with desirable commercial traits such as tolerance to excess light and heat stress, resistance to herbivore/pathogen, capacity to outcompete opportunistic organisms, or to express biosynthetic pathways into more productive strains (Fig. 3). Recent progress in genome sequencing, methagenome/metatranscriptome approaches, and genetic manipulation, yielded into significant advancement in microalgal research. In this paragraph, different strategies of genetic engineering, which revealed effective in improving algal productivity, are discussed. Moreover, additional solutions are proposed.

In the last decades, several efforts have been attempted to optimize the transformation efficiency of different microalgae species. Stable transformation was first developed in *C. reinhardtii*: being able to growth both in autotrophic and heterotrophic conditions, as haploid or diploid cells, *Chlamydomonas* was adopted as powerful genetic system for studying different physiological mechanisms. Nuclear transformation of *C. reinhardtii* may be achieved by several methods such as electroporation [120], *Agrobacterium*-mediated transformation [121], silicon carbide whiskers and positively-charged aminoclay nanoparticles [122, 123] and glass beads agitation method [124]. Both electroporation and *Agrobacterium*-mediated transformation also succeeded in transforming algae of economic interest such as *C. vulgaris*, *Neochloris oleoabundans* and *H. pluvialis* [125–127]. The introduction of foreign genes into the nuclear genome of microalgae is generally guided by a random integration event [128]. Nuclear gene expression is frequently subjected to strong silencing mechanisms due both to position effect and to epigenetic phenomena, similar to those of land plants [129]; indeed in microalgae, silenced multicopy transgenes exhibit high levels of DNA methylation as in land plants [130, 131].

In last years, many efforts have been attempted to increase the heterologous expression potential of microalgae. Increased expression of transgenes was obtained by fusing the sequences encoding the gene of interest and the selection marker in a unique bicistronic RNA [132, 133]. Recourse to a frequent subset of preferred codons results in elevated transcriptional levels, while the use of codons introducing unintended splicing signals negatively affects the overall expression of transgenes [134], thus a codon usage optimization is mandatory to maximize protein yield. To improve the selection of high-level expressing transformants, last-generation expression vectors exploit the 2A peptide [135] to generate transcriptional fusions between selection marker sequence (e.g. antibiotic resistance) and the gene of interest [132]. Transcription factors are gaining increasing attention as key regulators of metabolic pathways, in order to enhance yield of high-value molecules or to maximize the production of foreign proteins in microalgae. Over-expression of NsbZIP1, a transcription factor carrying the basic leucine zipper, resulted in both enhanced growth rate and higher lipid contents in *N. salina* [136].

The biolistic method (particle-gun bombardment) is the elective procedure for chloroplast transformation in microalgae [137]. The introduction of foreign genes into the plastome is guided by site-specific integration event (i.e. by homologous recombination). Expressing foreign genes in the chloroplast enables to circumvent gene silencing events, which affect the nuclear expression; moreover, it allows for the introduction of operons, encoding several enzymes of a pathway. However, the resulting transformants must undergo several rounds of selection in order to acquire the homoplasmic condition [138].

Further details concerning nuclear and chloroplast expression in *C. reinhardtii* are summarized in Table 2; moreover, many of these aspects have been covered extensively in a recent review [137].

**Table 2:** Transformation of *C. reinhardtii*

Organelle	DNA-delivery method	Genetic mechanism	Advantages	Disadvantages	Selection marker
Nucleus	Electroporation gene-gun bombarment *A. tumefaciens*-mediated Glass beads Silicon carbide whiskers and aminoclay nanoparticles	Ectopic recombination (random integration)	Protein can be expressed as secreted protein Post-translational modifications	Identification of high-expressing Transformants gene silencing	Resistance to Paromycin, Zeocin, Hygromycin, Chloramphenicol Auxotrophic complementation (*ARG7*, *NIT1*, *oee1*)
Chloroplast	Gene-gun bombarment Glass beads	Homologous recombination (site-specific integration)	Compartmentalization Lacks gene silencing High expression level	Need to identify homoplasmic transformant Lacks post-translational modifications	resistance to Spectinomycin Auxotrophic complementation (*atpB, psbH*)

This table displays DNA-delivery methods, genetic mechanism driving the transformation, and the most common selection markers employed so far. Major advantages and disadvantages of nuclear vs. chloroplast transformation are reported

Genome editing technology enables for both gene deletion and gene integration, therefore implementation of these novel genetic tools in microalgae would allow for manipulation of metabolic networks. Recently, a novel approach based on CRISPR–CAS9 genome editing technology have been successfully developed in both the marine diatom *P. tricornutum* [139] and in *C. reinhardtii*, allowing for deletion of specific gene functions [140]; the latter was achieved by a DNA-free CRISPR–Cas9 method and the outcome was the sequential *FTSY* and *ZEP* double-gene knockout, that resulted in improved photosynthetic productivity. Analogous approach was used to abolish the functions encoded by *MAA7*, *CpSRP43* and *ChlM* genes, which led to pale-green mutants [141]. In *C. reinhardtii*, the replacement of Cas9 with the Cpf1 ribonucleoprotein achieved a more efficient homology-directed DNA replacement [142]. However, a common limitation of free-DNA CRISPR–Cas9/Cpf1 methods resides in the lack of selection markers that, in turn, hinders a straightforward selection of the desired mutants; being the genome editing event induced at very low frequency (0.5–0.6%), a visible phenotype makes mutant selection easier. A DNA-based CRISPR–CAS9 method has been developed in the industrial oleaginous microalga *N. oceanica* [143] in which nuclear transformation can be efficiently performed by introducing expression cassettes obtained by PCR, making unnecessary the use of expression vectors [144]. Recently, a doubling of the lipid production in *N. gaditana* was obtained by deleting a transcription factor that acts as negative regulator in lipid biosynthesis [145].

As previously described, a truncated antenna size yielded into increased productivity in green microalgae (see “Promises of domestication by forward genetic in improving photosynthetic efficiency” section), thus proteins involved in the biogenesis of photosynthetic machinery can be targeted for increasing biomass production. Truncated light-harvesting antenna 1 (*TLA1*), a nuclear gene putatively involved in the regulation of the antenna size of *C. reinhardtii*, was up- and down-regulated by overexpression and RNAi, respectively. The strain over-expressing *TLA1* showed a larger antenna size for both photosystems and lower Chl *a*/*b* ration than the wild type, while its down-regulation resulted in the opposite phenotype changes [146]. LHCII, the nucleus-encoded light-harvesting proteins associated with PSII, tunes the light harvesting capacity to the prevailing light condition. In *C. reinhardtii*, LHCII translation efficiency is regulated by the cytosolic RNA-binding protein NAB1, which is subjected to specific nitrosylation in limiting light, thus making such repressor less-active and promoting accumulation of LHC [147].

Manipulation of RuBisCO activity, namely the major constraint for C assimilation e.g. under excess light conditions [148], may improve the photosynthetic yield [149]. Although site-directed mutants in the *rbcL* (RuBisCO large subunit) gene [150, 151] as well as hybrid variants with altered specificity of RuBisCO–RuBisCO activase interaction [152] have been generated, their over-expression in *C. reinhardtii* did not increase the overall photosynthetic yield.

Recently, biomass productivity as well as lipid yield increased up to 40% in the oleaginous *Nannochloropsis oceanica* by overexpressing endogenous RuBisCO activase [153]. Conversely, a reduction in the RuBisCO activity by site directed mutagenesis resulted in a ten-fold higher H_2_ production in *C. reinhardtii* [154], likely because Calvin-Benson cycle competes with Hydrogenase for reducing equivalents.

Further strategies included (i) the PCR-based gene shuffling of *Chlamydomonas* rbcL with sequences representing natural variants of this gene, which yielded isoforms with higher *V*_max_ of carboxylation catalysis [155]; (ii) regulation of RuBisCO accumulation according to culture conditions, by tuning mRNA level of the nuclear maturation factor MRL1 [156]; (iii) overexpression of. sedoheptulose 1,7-bisphosphatase from *C. reinhardtii*, which succeeded in enhancing photosynthetic efficiency in *D. bardawil* [157]; finally, the over-expression of Low-CO_2_ Inducible (LCI) proteins in *C. reinhardtii,* under conditions which typically repress their synthesis (i.e. high CO_2_ concentration), increased biomass production under elevated CO_2_ conditions as much as 80% than control strain [158].

High productivity in open ponds is restricted to species which adapted to high salt concentration (e.g. *Dunaliella*) or high pH (e.g. *Spirulina*), thus outcompeting naturally occurring contaminants. Hence, a trait which confers competitive advantage over undesirable microorganisms, is crucial both to increase the biomass productivity and to reduce the operating costs for maintenance of axenic cultures (Fig. 1), particularly in either open pond or heterotrophic conditions. In this perspective, non-canonical substrates may be employed for sustaining algal growth. The expression of the phosphite dehydrogenase D (PTXD) from *P. stutzeri* WM88 [159] confers to *C. reinhardtii* the capacity of metabolizing phosphite, namely a P source which cannot be utilized by plants, fungi and most bacteria. Transgenic *Chlamydomonas* cells showed higher fitness than *S. obliquus* in competition experiments in which phosphite-repleted/phosphate-depleted medium was used [160].

Some algal species are strict autotrophs or are highly selective for their C source (e.g. *Chlamydomonas* for acetate), thus trophic conversion by metabolic engineering would be desirable. *Chlamydomonas* cells expressing the hexose transporter HUP1 (monosaccharide-H^+^ symporter from *C. kessleri*) metabolized externally supplied glucose for heterotrophic growth and showed higher H_2_ production capacity; however, results suggest that glucose cannot fully replace acetate as a C source, for long-term growth in the dark [161].

Other algal species can metabolize a large array of sugars, and strongly increase their productivity under heterotrophic or mixotrophic growth conditions [162]; however, heterotrophic growth requires additional costs due to need for exogenous carbon source and maintenance of axenic conditions. Algal strains able of metabolizing raw lignocellulosic biomass scraps, namely cheap agricultural wastes, would certainly contribute to make the whole process economically viable. Foreign genes encoding bacterial and fungal plant Cell Wall Degrading Enzymes (CWDEs), were constitutively expressed in microalgae and addressed to the secretory pathway [132]. In *C. reinhardtii*, yield of secreted proteins was improved up to eight-fold by fusing both the putative signal peptide of gametolysin and the repeated serine-proline module, to the N and C terminus of the recombinant protein, respectively [163]. Contrary to plant cell, some species of unicellular green algae possess a cell wall mainly constituted by proteins (e.g. *C. reinhardtii*) [164], thus lack of polysaccharides as major components circumvents the deleterious effects of expressing CWDEs in plants, likely related to hyper-immune responses [165, 166]. Although some algal spp. synthesizes endogenous CWDEs [167], the native cellulolytic machinery is not efficient enough for degrading hydrolysis-recalcitrant substrates such as lignocellulose. Thus, a promising perspective is the expression of a range of secreted CWDEs, including polygalacturonases, hemicellulases, cellulases and ligninases in a unique algal culture, analogously to the approaches developed in yeasts which yielded into strains able to grow on cellulosic substrates [168].

**Fig. 4:**
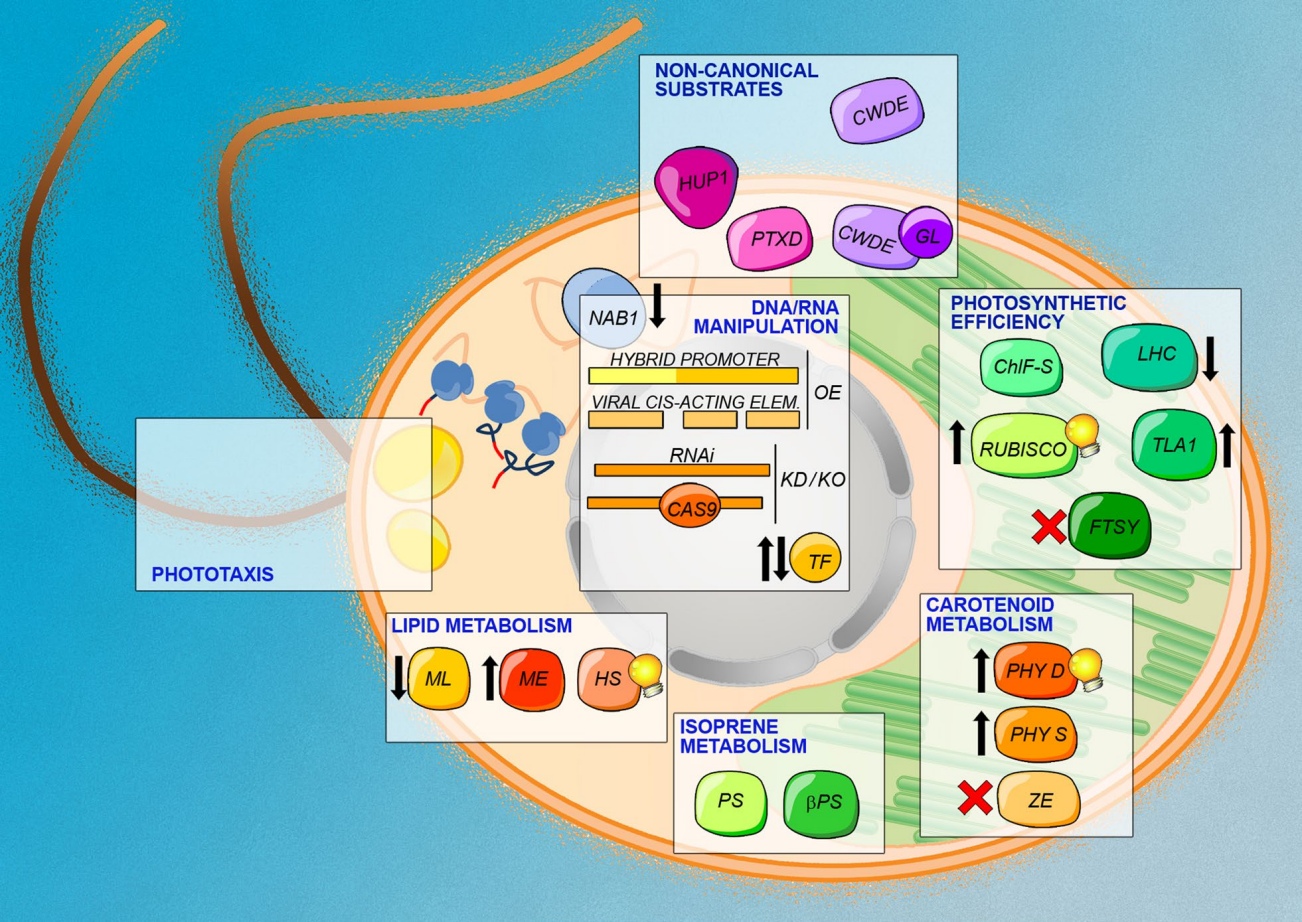
Potential traits to be implemented in GM—*C. reinhardtii* cell. The diagram displays a number of genetic strategies, aimed to enhance productivity in mass culture of microalgae. Gene over-expression (OE) using hydrid promoters or viral cis-acting elements and gene disruption/down-regulation (KO/KD) by Crispr–Cas9 and RNAi approaches are indicated. Some traits that may result in higher productivity include an increased photosynthetic efficiency, improved phototaxis, the use of non-canonical substrates, and optimized carotenoid, lipid and isoprene metabolism. Up- and down-ward pointing arrow mean up- and down-regulation, respectively, and are referred to the expression level of the corresponding endogenous enzyme. Bulb and red cross mean enzymatic in vitro improvement and loss of function, respectively. Abbreviations: *Chl-f S* chlorophyll *f* synthase, *CWDE* cell-wall degrading enzyme, *FTSY* chloroplast signal recognition particle, *GL* gametolysin signal peptide, *HS* hydrocarbon-synthase, *HUP1* hexose-proton symporter, *LHC* light harvesting complexes, *ME* malate dehydrogenase, *ML* multifunctional lipase, *NAB1* RNA-binding protein, *PHY D* phytoene desaturase, *PHY S* phytoene synthase, *PS* patchoulol synthase, *PTXD* phosphite dehydrogenase, *β-PS* β-phellandrene synthase, *TF* transcription factor, *TLA1* truncated light-harvesting antenna 1, *ZEP* zeaxanthin epoxidase

An overview of the major genetic manipulations which may lead to an improvement of biomass productivity is represented in Fig. 4.

## Conclusions

Commercially cultivated for several decades, microalgae are now recognized to offer a great potential for exploitation in different fields including pharmaceuticals, aquaculture and renewable energies. Former efforts in their industrial applications mainly focused in optimizing culture parameters and selecting the best performing wild type strain. However, to promote cultivation of microalgae as a new biotechnological sector, a number of challenges still have to be overcome. Domestication strategies achieved by genetic and metabolic engineering will be crucially important to isolate “*smart strains*” with improved yield, in order to make the production successfully marketed. The opportunities offered by investments in both basic and applied research, are considerable: (i) the rapid evolution of genome sequencing techniques will help defining the gene networks controlling growth, while -omics approaches allow to identify regulatory points of cellular pathways, thus enabling manipulation of key metabolic steps; (ii) prospective redesigns of algal system include light-to-biomass conversion efficiency, oil content/composition, nutrient recovery capacity; (iii) extend the genetic transformation techniques, now carried out successfully in few species only, to the most industrially-relevant species, will offer the opportunity to address the biological constraints limiting growth yield; finally, (iv) the development of reproducible genome editing techniques will permit a fine matching of the primary metabolism to the mass culture conditions, or the development of molecular strategies for strain containment. Encouraging results have recently been obtained by boosting light-use-efficiency or by strengthening specific metabolic pathways. Additional research efforts and funding for implementing innovative biorefineries, will realistically support progress toward next-generation algal biotechnology.

## Abbreviations

Chl: chlorophylls
CWDE: cell wall degrading enzyme
DHA: docosahexaenoic acid
DW: dry weight
EMS: ethyl methanesulfonate
EPA: eicosapentaenoic acid
GMO: genetically-modified organism
lc-PUFA: long-chain polyunsaturated fatty acid
LHCBM/LHCSR: light-harvesting complex proteins
NGT: *N*′-nitro-*N* nitrosoguanidine
NPQ: non-photochemical quenching
OD: optical density
PAR: photosynthetically active radiation
PBR: photobioreactor
PSII: photosystem II
ROS: reactive oxygen species
RuBisCO: ribulose 1,5-bisphosphate carboxylase-oxygenase
RuBP: ribulose 1,5-bisphosphate
TAGs: triglycerides
TW-y: terawatts-year
